# Evidence of Impaired Neuroimmune System in Post‐COVID Syndrome—A Whole Brain Magnetic Resonance Spectroscopy Study

**DOI:** 10.1002/jmv.70762

**Published:** 2025-12-22

**Authors:** Ann‐Katrin Hennemann, Nima Mahmoudi, Katja Döring, Heinrich Lanfermann, Karin Weissenborn, Meike Dirks, Xiao‐Qi Ding

**Affiliations:** ^1^ Department of Neurology Hannover Medical School Hannover Germany; ^2^ Institute of Diagnostic and Interventional Neuroradiology Hannover Medical School Hannover Germany

**Keywords:** cognitive function, follow‐up, neuroimmune system, post‐COVID, whole brain MRS

## Abstract

Chronic fatigue, mood disturbances, and cognitive deficits characterize the neurological post‐COVID syndrome (PCS). This study aimed to find out if PCS shows a diagnostic brain metabolic pattern that might also support clarification of the PCS pathology. Whole brain proton magnetic resonance spectroscopy imaging (wbMRSI) was applied to assess brain metabolites in PCS patients. Patients' data were compared to those of matched healthy controls examined before the COVID pandemic. Patients underwent clinical and neuropsychological assessment and filled in self‐report questionnaires related to fatigue, mood, and health‐related quality of life. Thirty PCS patients were enrolled into the study. WbMRSI showed significantly reduced levels of brain myo‐inositol, which is considered as representative of astrocyte and microglia activity, in the frontal, temporal and parietal lobes, bilaterally, and in the cerebellum of the patients compared to controls. Patients' creatine was higher in the left frontal lobe and the combined glutamate/glutamine peak was lower in the right parietal lobe. *N*‐acetyl‐aspartate, an indicator of neuronal integrity, as well as choline that reflects cell membrane turnover, showed no group differences. The findings suggest an alteration of the neuroimmune system in PCS patients, without indication of disturbed neuronal integrity or alteration of the cerebral energy metabolism.

## 1 Introduction

About 5 years after the severe acute respiratory syndrome coronavirus‐2 (SARS‐CoV‐2) outbreak with more than 775 million confirmed infections [1] and more than 7 million deaths, the focus in patient care and research has moved from the analysis of the acute phase of the disease (COVID‐19) to the long‐term sequelae. Several studies indicate that about 10% of those who suffered COVID‐19 develop a post‐COVID syndrome (PCS), which is diagnosed in case of persistent or newly developed clinical symptoms, such as fatigue or cognitive dysfunction, more than 3 months after a SARS‐CoV‐2 infection if other possible causes are excluded [2, 3]. The most frequent feature of PCS is characterized by chronic fatigue, attention, concentration and memory deficits, difficulties finding words, mental and physical exhaustibility, sleep disturbances and headaches, as well as depression, anxiety, and reduced quality of life [4]. So far, the pathophysiological mechanisms behind the syndrome are unclear. Sustained virus replication within the brain, altered systemic and neuronal immune response toward the virus or relative hypoxia due to endothelial damage of the cerebral microvasculature and widespread microthrombosis are discussed as are maladaptive coping strategies toward the adverse aftermaths of COVID‐19 [5].

In order to facilitate the diagnosis and to develop treatment options, efforts are made worldwide to detect biomarkers such as a diagnostic blood cytokine or immune cell pattern or diagnostic findings in brain imaging or cerebrospinal fluid (CSF).

From early on in the SARS‐CoV‐2 pandemic, studies have looked for structural, functional, or metabolic alterations of the brain [6, 7]. Now, in the postacute phase, brain imaging studies can be performed more easily and in more detail. An increasing number of studies present, for example, magnetic resonance imaging (MRI) or positron emission tomography (PET) findings in patients with PCS compared to participants, who recovered from the infection, or healthy controls. Studies on patients with PCS report altered connectivity of brain structures [8, 9], changes of cortical thickness and volume [10], altered water content and diffusivity [8, 11], hypoperfusion [8, 12], and decreased glucose metabolism especially in the frontal, parietal, and temporal cortex [7, 13]. Interestingly, the alterations are more pronounced in the right than in the left hemisphere irrespective of the method applied [8, 12, 14, 15].

Diez‐Cirarda and colleagues examined 84 patients with cognitive deficits as part of the PCS about 11 months after SARS‐CoV‐2 infection. MRI findings revealed hippocampal gray matter atrophy together with altered microstructural integrity, hypoperfusion, and functional connectivity changes compared to healthy controls [8]. Heine and colleagues recruited 50 patients with neurological PCS about 7.5 months after SARS‐CoV‐2 infection [9]. Diffusion tensor imaging (DTI) analyses showed aberrant fractional anisotrophy of the thalamus. Additionally, shape deformations and decreased volumes of the left thalamus, putamen, and pallidum were observed. Ajcevic et colleagues examined cerebral perfusion with MRI technique in 24 PCS patients with subjective cognitive complaints [12]. They observed regional cerebral hypoperfusion in widespread cerebral networks, especially in the frontal cortex, but also the parietal and temporal cortex. Recently, Besteher and colleagues identified cortical thickness increase in PCS patients with cognitive impairment and also a distinct immunophenotype in this patient group [16].

Data acquisition in the mentioned studies took place between 3 and 11 months after the SARS‐CoV‐2 infection. This impedes comparison and interpretation of the findings.

Magnetic resonance spectroscopy imaging (MRSI) offers a noninvasive means to quantify brain metabolites that reflect gliosis (e.g., myo‐inositol), neuronal integrity (e.g., *N*‐acetylaspartate), and energy metabolism (e.g., creatine, choline), and thus may provide crucial insights into the underlying mechanisms of PCS. A recent study by Ernst and colleagues reported altered metabolite levels in selected brain regions of PCS patients using single‐voxel MRS, suggesting astroglial activation and neuronal dysfunction [17]. While these findings have highlighted the potential involvement of neuroimmunologic alterations in the pathophysiology of PCS, particularly in patients reporting persistent cognitive and neuropsychiatric symptoms (e.g., brain fog, fatigue, depression), the results are limited by the used technical method: single‐voxel measurements from selected small brain regions, thus information about the PCS‐related metabolic status within the whole brain are missing. Therefore, we carried out the present study with the newly established whole‐brain MRSI (wbMRSI) allowing to estimate brain metabolite distributions within the whole brain with high spatial resolution under standardized acquisition conditions, and to reveal PCS‐related brain metabolic disorder [16]. This methodological advance enables a more systematic investigation of regional metabolic alterations and their potential link to PCS symptoms. Therefore, our work contributes novel, high‐resolution data to a still developing field and addresses current gaps in the neuroimaging literature on post‐COVID sequelae.

## 2 Methods

### 2.1 Study Design and Cohort

Fourty‐seven patients with PCS who had undergone clinical examination and neuropsychological assessment at the Department of Neurology at Hannover Medical School (MHH), Hannover, Germany, underwent wbMRSI at the Institute for Diagnostic and Interventional Neuroradiology at MHH. Inclusion criteria were positive polymerase chain reaction (PCR) test for SARS‐CoV‐2 and persisting symptoms typical for PCS for more than 3 months after the infection. Exclusion criteria were age below 20 or above 70 years, any other systemic diseases that might significantly affect brain structure and function, medication affecting the central nervous system as well as contraindications against MRI.

To exclude possible interference with PCS, patients with medically controlled hypertension and patients with obesity (body mass index (BMI) > 30) were excluded from the final data analysis leaving data of 30 patients for further analysis.

The patients' MRS data were compared to those of 30 healthy controls matched for sex, age, and BMI who had been examined before the pandemic in a study aimed at the elaboration of norm data [18, 19]. Moreover, metabolite levels of patients with and without distinct cognitive impairments were compared between each other as well as with those of the controls.

### 2.2 Ethics

This study was carried out in accordance with the Declaration of Helsinki. All participants included in the study gave written informed consent. The study was approved by the Local Ethics Committee (Nr. 10093_B0_S_2021).

### 2.3 Clinical Assessment

All patients underwent a structured anamnesis and a neurological examination as well as psychometric testing. In addition, the patients were asked to fill in self‐report questionnaires addressing fatigue (Fatigue Impact Scale, FIS) [20], mood (Hospital Anxiety and Depression Scale, HADS) [21], and health‐related quality of life (SF‐36) [22]. The structured anamnesis covered the characteristic clinical symptoms of PCS: olfactory/taste disorder, fatigue, insomnia, concentration deficits, memory impairment, difficulties in finding words, headache, myalgia, and paresthesia.

The neurocognitive assessment included the Montreal Cognitive Assessment (MoCA) [23], the Word‐Figure‐Memory‐Test (WFMT) [24], the Recurring‐Figures‐Test (RFT) [25], the D2 Test of Attention [26], and the subtests “alertness,” “divided attention,” “flexibility,” and “covert shift of attention” from the computer‐based test battery for the assessment of attention (TAP) [27].

### 2.4 MR and wbMRSI Examinations

All participants' MR data were acquired using a 12‐channel phased‐array head coil at 3 T (Verio, Siemens, Erlangen, Germany). All sequences were acquired with the same angulation. The MRI protocol consisted of T1‐weighted (T1w) and T2‐weighted (T2w) sequences, a T2w‐fluid attenuation inversion recovery (FLAIR) sequence, a 3D‐T1‐w MPRAGE (magnetization‐prepared rapid gradient echo) sequence with isotropic resolution (1 × 1 × 1 mm), and a volumetric spin‐echo planar spectroscopic imaging (EPSI) sequence for wbMRSI (repetition time (TR)/echo time (TE) = 1550/17.6 ms, 50 × 50 voxels in‐plane and 18 slices, field of view (FOV) 280 × 280 × 180 mm^3^) [28]. Auto‐shimming was manually adjusted for reducing spectral linewidth and to improve the magnetic field inhomogeneity. An additional EPSI sequence was conducted to get MRSI data without the suppression of water signals for various processing tasks and served as an internal reference for normalizing the metabolite concentrations. To complement the wbMRSI data, DTI and additional multiparametric MRI sequences were acquired as part of the imaging protocol, providing a basis for future investigations into structural and functional correlates of post‐COVID brain alterations.

### 2.5 Analysis of MR and MRSI Data

Neuroradiologists examined the MR images beforehand to identify incidental findings and structural abnormalities.

The EPSI data, along with the MPRAGE data, were analyzed using the software package Metabolic Imaging and Data Analysis System (MIDAS), as previously described [18, 29]. This analysis was performed to create brain maps of the metabolites NAA, Cho, tCr, Glx, mI, and the corresponding spectral linewidth (LW). Additionally, maps of the relative CSF component were derived. Subsequently, all the maps underwent spatial transformation and interpolation to a uniform spatial reference with a 2 mm isotropic resolution. Thereafter, the spatial reference was linked to an atlas that delineated eight brain lobes, that is, the frontal lobe left (LFL) and right (RFL), the temporal lobe left (LTL) and right (RTL), the parietal lobe left (LPL) and right (RPL), the occipital lobe left (LOL) and right (ROL), and the cerebellum, where lobe‐based analyses were conducted similarly as in previous research projects [30].

Based on the derived corresponding brain maps the regional metabolite concentrations [NAA], [Cho], [tCr], [Glx], and [mI] were determined in institutional units (i.u., = ratios to internal water, and expressed as mean ± SD) for each of the nine brain regions (regional metabolite concentration) of each patient, as well as the mean LW and the mean fractional volume of the CSF (FVCSF). To avoid partial volume effects and for data quality assurance the regional metabolite concentrations were corrected for CSF volume contribution according to the relation *Met*′ = *Met*/(1 – *f*_csf_) for 0 < *f*_csf_ < 0.3, where Met is the uncorrected metabolite value and *f*_csf_ is the FVCSF in the MRSI voxel, while voxels with a spectral LW > 12 Hz or a voxel with *f*_csf_ > 0.3 were excluded from the calculation to minimize partial volume effects and spectral distortion applications [29]. To provide an overview about the brain metabolite distributions, mean value metabolite maps calculated from the corresponding maps of the patients as well as from those of the healthy controls were also derived.

The MRSI data of the healthy controls were obtained before the SARS‐CoV‐2 pandemic using the same MR scanner and the same imaging protocol, where the regional metabolite concentrations as well as the LW and FVCSF were determined in the same manner [18, 19].

### 2.6 Statistical Analysis

Clinical data and the results of questionnaires and psychometric tests were checked for normal distribution by the Kolmogorov–Smirnov test. Not normally distributed data are shown as median and 25th/75th percentile, normally distributed as mean ± SD.

After tests for normality with the Shapiro–Wilk test and *Q*–*Q* plots, the brain regional metabolite concentrations and the spectral linewidth measured from patients were compared to those of the matched healthy controls by using the paired sample *t*‐test.

For patients' metabolite concentrations that showed significant alterations, the Spearman rank test was used to estimate their possible correlations to the neuropsychological findings, and an analysis of covariance (ANCOVA) including age and sex as covariates and post hoc tests with Bonferroni correction for multiple comparisons was used to look for possible differences between patient groups with and without defined complaints or cognitive deficits and the controls.

Statistical tests were two‐tailed, the significance level was set at *p* < 0.05. Statistical analyses were performed using SPSS version 28 (SPSS IBM, New York, USA).

## 3 Results

### 3.1 Clinical Assessment

Both the patient and control group consisted of 20 women and 10 men, respectively, with a mean age of 39 years and a mean BMI below 25 (Table 1). The timespan between SARS‐CoV‐2 infection and MRS was 14.2 months in median. The psychometric testing had been performed in median 1 month before MRS. All but one of the patients had a mild to moderate course of COVID‐19, not requiring hospitalization. The one who had been referred to the hospital had not needed intensive care therapy. In total, 90% of the patients complained about chronic fatigue and deficits in concentration, 80% about memory impairment and 50% about difficulties in finding words. One third reported headache and sleep disturbances, 23% myalgia, and 17% paresthesia. Three patients still suffered from loss of smell and taste. There was no difference in the symptom pattern between male and female patients.

**Table 1:** Baseline characteristics of the whole patient cohort and healthy controls.

	Healthy controls (*n* = 30)	Entire patient cohort (*n* = 30)	Female (*n* = 20)	Male (*n* = 10)	*p* (controls vs. entire patient cohort)	*p* (female vs. male)
Age (years)	38.7 (±11.8)	39.1 (±11.3)	42.8 (±11.1)	31.9 (±7.9)	0.894	0.010
Timespan positive PCR to MRS date in months	—	14.2 (9.7/17.0)	13.2 (10.7/16.2)	11.6 (8.6/12)		0.692
Timespan psychometric assessment to MRS date in months	—	1.1 (0.6/1.8)	1.4 (0.4/2.9)	0.8 (0.6/1.2)		0.235
Years of education	—	13 (10/13)	13 (10/13)	13 (11.5/13)		0.451
Mean BMI (kg/m^2^)	23.5 (±3.0)	24 (±3.1)	24 (±3.3)	24 (±2.7)	0.563	0.685
Infection severity requiring hospitalization	—	1 (3.3%)	1 (5%)	0 (0%)		
*Clinical symptoms*						
Olfactory/taste disorder		3 (10%)	1 (5%)	2 (20%)		
Fatigue		27 (90%)	18 (90%)	9 (90%)		
Insomnia		10 (33.3%)	6 (30%)	4 (40%)		
Deficits in concentration		27 (90%)	18 (90%)	9 (90%)		
Memory impairment		24 (80%)	16 (80%)	8 (80%)		
Difficulty in finding words		15 (50%)	9 (45%)	6 (60%)		
Headache		10 (33.3%)	7 (35%)	3 (30%)		
Myalgia		7 (23.2%)	4 (20%)	3 (30%)		
Paresthesia		5 (16.7%)	3 (15%)	2 (20%)		

*Note:* Results expressed as mean ± SD or median and 25th/75th percentiles. *T*‐test between healthy controls and the entire patient cohort and between female and male patients for age and mean BMI, Mann–Whitney *U* test for years of education and timespan positive PCR to MRS date and timespan psychometric assessment to MRS date.

Abbreviations: BMI, body mass index; SD, standard deviation.

In accordance with the patients' complaints, the median FIS score of 101 (norm < 40) indicated disabling fatigue and the median SF‐36 scores reduced physical and mental health‐related quality of life in the patients (Table 2). Of note, none of the patients had a FIS within the normal range. HADS anxiety and depression scores were in median within the normal range; however, nine patients had an abnormal depression score and five an abnormal anxiety score (> 10).

**Table 2:** Baseline data from the self‐report questionnaires of the patient cohort (*n* = 30).

Self‐report questionnaires	Median (25th/75th percentile)	Number of abnormal test results
FIS	101 (89/125)	30/30 (100%)
HADS depression score	7 (5/12)	9/30 (30%)
HADS anxiety score	7 (4/10)	5/30 (17%)
SF‐36 Physical Component Summary Score	33.13 (28.18/42.24)	14/30 (47%)
SF‐36 Mental Component Summary Score	40.00 (28.31/47.16)	10/30 (33%)

*Note:* Results expressed as median and 25th/75th percentiles. Data are also presented as number of abnormal test results.

Abbreviations: FIS, Fatigue Impact Scale; HADS, Hospital Anxiety and Depression Scale.

Table 3 shows the psychometric test results. The median MoCA score was 26; eight patients achieved an abnormal score (< 26). Also, eight patients showed abnormal memory function in the recognition of either words and/or figures in the WFMT (*z*‐score < −1.3), while only two achieved abnormal results in the RFT (≤ 10th percentile) (data not shown). The performance of the D2 Test of Attention was abnormal regarding the percentage of errors in 5 and regarding the number of assessed items in 4 of 27 patients (≤ 10th percentile).

**Table 3:** Results from the psychometric test battery of the patient cohort.

Psychometric test battery	Median (25th/75th percentile)	Number of abnormal test results
MoCA (*n* = 30)	26.0 (25.0/29.0)	8/30 (27%)
WFMT words *z*‐score (*n* = 30)	−0.551 (−1.096/0.701)	6/30 (20%)
WFMT figures *z*‐score (*n* = 30)	−0.312 (−0.780/0.629)	5/30 (17%)
		
		
D2 Test of Attention errors % (*n* = 27)	5.2 (2.1/12.6)	5/27 (19%)
D2 Test of Attention items‐errors (*n* = 27)	398.0 (300.0/496.0)	4/27 (15%)
*Test battery for the assessment of attention (TAP)* a		
Alertness		
Reaction time, without warning	262 (243/331) (*n* = 29)	13/30 (43%)
Reaction time, with warning	284 (232/345) (*n* = 29)	17/30 (56%)
Phasic alertness	−0.013 (−0.081/0.073) (*n* = 29)	7/30 (23%)
Flexibility		
Reaction time	822 (685/1035) (*n* = 28)	6/30 (20%)
Errors	2.0 (1.0/8.5) (*n* = 28)	7/30 (23%)
Divided attention		
Reaction time, auditory stimuli	678 (691/880) (*n* = 28)	18/30 (60%)
Reaction time, visual stimuli	779 (691/880) (*n* = 28)	4/30 (13%)
Errors	0.5 (0.0/2.0) (*n* = 28)	4/30 (13%)
Misses	1.5 (0.3/4.0) (*n* = 28)	10/30 (33%)
Covert shift of attention		
Valid hint left reaction time	344 (298/401) (*n* = 27)	12/29 (41%)
Valid hint right reaction time	323 (293/390) (*n* = 27)	10/29 (34%)
Invalid hint left reaction time	392 (317/448) (*n* = 27)	12/29 (41%)
Invalid hint right reaction time	413 (353/464) (*n* = 27)	16/29 (55%)

Abbreviations: MoCA, Montreal Cognitive Assessment; WFMT, Word‐Figure‐Memory‐Test.

^a^ The raw data of the TAP represent only those patients who were able to complete the test. If a patient was unable to perform the test, for example, because of disabling fatigue or increasing concentration deficits, the result was scored abnormal.

Regarding reaction times as well as errors and misses about half of the patients achieved abnormal results in the alertness test (wakefulness), the divided attention test, and the covert shift of attention test (tests of selective visuospatial attention), while only about 20% achieved abnormal results in the flexible reaction time test, a test of focused attention and cognitive flexibility.

### 3.2 Results of wbMRSI Measurements

**Figure 1:**
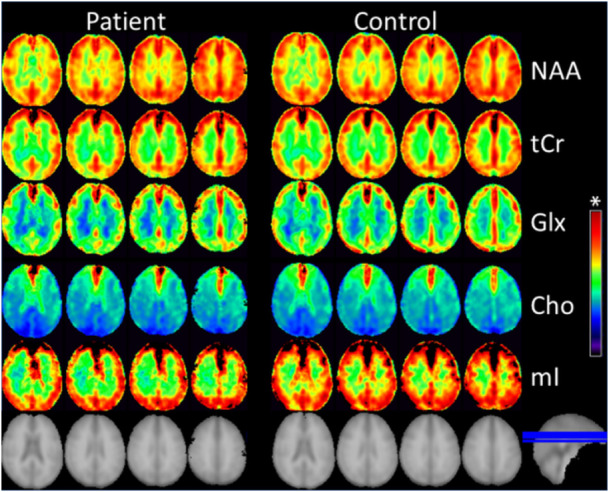
Example mean value maps of the metabolites NAA, tCr, Glx, Cho, and mI, averaged among the corresponding maps of the patients (left part) and of the controls (right part). The corresponding anatomic images are also shown. *Colored bar showing the intensity range with internal unit (i.U.) determined in ratio to brain internal water: bottom to top represents from 0 up to 18 i.U. for *N*‐Acetyl‐Aspartate (NAA), creatine (tCr), and glutamate/glutamine (Glx), and to 5.5 i.U. for choline (Cho) and myo‐inositol (mI).

Example brain maps of mean value for brain metabolites NAA, tCr, Glx, Cho, and mI—averaged among those of the patients (left part) and those of the controls (right part)—are shown in Figure 1. The mean value mI maps of the patients revealed weaker signal intensity than those of the controls within larger brain areas, without such clear signal intensity differences on the mean value maps of other metabolites between patients and controls. This indicates a reduction of mI concentration in most brain regions of the patients.

The metabolite concentrations in different brain regions measured by wbMRSI are shown in Table 4. In contrast to NAA, Cho, tCr, and Glx myo‐inositol showed widespread significant differences between patients and controls; that is, mI levels were significantly lower in the patients than in controls in bilateral frontal, parietal, and temporal lobes, and in the cerebellum. The difference was more pronounced on the right side than on the left. In addition, tCr in the left frontal lobe was significantly higher and Glx in the right parietal lobe was significantly lower in the patients than in the controls. NAA and Cho showed no group differences. A further parameter that showed significant group differences in multiple brain regions was the spectral linewidth, which was significantly broader in patients than in controls in bilateral frontal, temporal, and parietal lobes, but not the occipital lobes and the cerebellum (Table 4). After Bonferroni correction for multiple comparisons group differences remained significant for mI levels in the right frontal, temporal, and parietal lobe and the cerebellum, and for linewidth in the left frontal, left parietal, and both temporal lobes.

**Table 4:** Regional metabolite concentration in different brain regions measured by wbMRS.

Regions	Metabolite	Patients (*n* = 30)	Controls (*n* = 30)	*p*
*N‐acetyl‐aspartate*				
RFL		11.95 ± 0.89	11.88 ± 0.84	0.726
LFL		12.07 ± 0.84	11.91 ± 0.86	0.412
RTL		10.22 ± 0.89	10.05 ± 0.81	0.306
LTL		10.88 ± 0.70	10.62 ± 0.83	0.140
RPL		12.08 ± 0.75	12.23 ± 0.75	0.401
LPL		12.35 ± 0.68	12.29 ± 0.70	0.747
ROL		12.10 ± 0.75	11.89 ± 0.83	0.305
LOL		12.23 ± 0.67	12.25 ± 0.79	0.905
CBL		10.43 ± 0.55	10.14 ± 0.75	0.114
*Choline*				
RFL		2.29 ± 0.20	2.31 ± 0.18	0.751
LFL		2.38 ± 0.26	2.32 ± 0.18	0.298
RTL		1.98 ± 0.16	1.96 ± 0.14	0.658
LTL		2.13 ± 0.17	2.11 ± 0.18	0.697
RPL		1.90 ± 0.18	1.96 ± 0.17	0.105
LPL		2.02 ± 0.21	2.01 ± 0.19	0.886
ROL		1.56 ± 0.14	1.57 ± 0.13	0.633
LOL		1.65 ± 0.16	1.64 ± 0.14	0.703
CBL		2.74 ± 0.24	2.75 ± 0.20	0.930
*Creatine*				
RFL		11.49 ± 0.75	11.25 ± 0.58	0.217
LFL		11.81 ± 0.91	11.35 ± 0.70	**0.037**
RTL		10.01 ± 0.57	9.99 ± 0.59	0.875
LTL		10.88 ± 0.85	10.64 ± 0.67	0.259
RPL		10.67 ± 0.65	10.87 ± 0.70	0.259
LPL		11.11 ± 0.81	10.95 ± 0.70	0.393
ROL		10.65 ± 0.72	10.70 ± 0.78	0.791
LOL		11.13 ± 0.72	11.15 ± 0.63	0.918
CBL		14.32 ± 1.28	14.18 ± 1.39	0.716
*Glutamine/glutamate*				
RFL		8.96 ± 0.85	9.06 ± 0.67	0.586
LFL		9.47 ± 0.85	9.46 ± 0.68	0.961
RTL		8.59 ± 0.88	8.43 ± 0.82	0.404
LTL		9.27 ± 0.89	9.01 ± 0.69	0.197
RPL		8.51 ± 0.76	8.87 ± 0.56	**0.013**
LPL		8.91 ± 0.81	9.15 ± 0.72	0.250
ROL		8.49 ± 0.66	8.58 ± 0.74	0.568
LOL		8.75 ± 0.97	8.67 ± 0.59	0.694
CBL		9.79 ± 0.70	9.69 ± 0.51	0.517
*Myo‐Inositol*				
RFL		3.49 ± 0.65	3.97 ± 0.59	**0.006**
LFL		3.96 ± 0.66	4.28 ± 0.60	0.056
RTL		3.59 ± 0.47	3.99 ± 0.51	**0.006**
LTL		4.16 ± 0.64	4.48 ± 0.53	**0.037**
RPL		3.25 ± 0.56	3.76 ± 0.46	**0.001**
LPL		3.52 ± 0.46	3.85 ± 0.53	**0.012**
ROL		3.69 ± 0.42	3.76 ± 0.51	0.565
LOL		3.85 ± 0.41	3.94 ± 0.51	0.478
CBL		4.14 ± 0.51	4.70 ± 0.61	**0.001**
*Spectral linewidth*				
RFL		7.63 ± 0.50	7.33 ± 0.44	**0.013**
LFL		7.63 ± 0.59	7.25 ± 0.41	**0.003**
RTL		7.87 ± 0.49	7.52 ± 0.43	**0.008**
LTL		7.78 ± 0.52	7.28 ± 0.42	**0.001**
RPL		7.19 ± 0.82	6.72 ± 0.66	**0.023**
LPL		7.23 ± 0.81	6.59 ± 0.65	**0.002**
ROL		6.77 ± 0.77	6.69 ± 0.62	0.666
LOL		6.67 ± 0.61	6.55 ± 0.59	0.443
CBL		7.49 ± 0.55	7.49 ± 0.49	0.993
*Cerebrospinal fluid*				
RFL		0.0796 ± 0.0083	0.0776 ± 0.0098	0.320
LFL		0.0804 ± 0.0100	0.0798 ± 0.0098	0.797
RTL		0.0616 ± 0.0142	0.0598 ± 0.0152	0.590
LTL		0.0695 ± 0.0161	0.0688 ± 0.0155	0.852
RPL		0.0885 ± 0.0138	0.0890 ± 0.0156	0.886
LPL		0.0872 ± 0.0138	0.0924 ± 0.0135	0.120
ROL		0.0659 ± 0.0170	0.0628 ± 0.0133	0.387
LOL		0.0653 ± 0.0191	0.0645 ± 0.0159	0.854
CBL		0.0694 ± 0.0092	0.0711 ± 0.0067	0.403

*Note:* Results for patients and controls are expressed as mean ± SD. Paired sample *t*‐test was used. Bold values indicate statistically significant.

Abbreviations: BL, cerebellum; LFL, left frontal lobe; LOL, left occipital lobe; LPL, left parietal lobe; LTL, left temporal lobe; RFL, right frontal lobe; ROL, right occipital lobe; RPL, right parietal lobe; RTL, right temporal lobe; SD, standard deviation.

### 3.3 Variation of Altered Myo‐Inositol Levels Related to Neuropsychological Findings in PCS Patients

**Figure 2:**
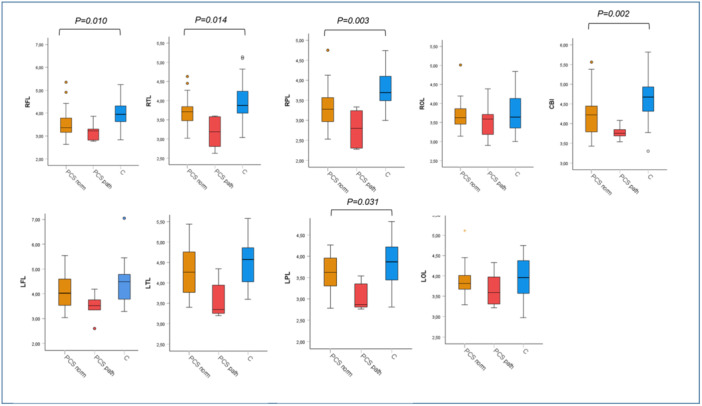
Myo‐inositol level in PCS patients drawn according to results in the Word‐Figure‐Memory‐Test (category recognition of figures *z*‐scores) and the values of the controls in different brain regions. Myo‐inositol (mI) level categorized for post‐COVID syndrome (PCS) patients with normal values (PCS norm, orange, *n* = 25) in Word‐Figure‐Memory‐Test (WFMT) recognition of figures *z*‐scores, pathological values (PCS path, red, *n* = 5) and the values of the control group (C, blue, *n* = 30). ANCOVA and post hoc test were used for statistical analysis. CBL, cerebellum; LFL, left frontal lobe; LOL, left occipital lobe; LPL, left parietal lobe; LTL, left temporal lobe; RFL, right frontal lobe; ROL, right occipital lobe; RPL, right parietal lobe; RTL, right temporal lobe.

**Figure 3:**
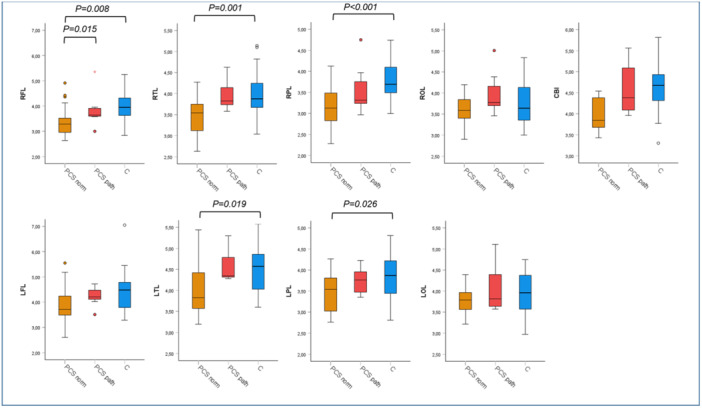
Myo‐inositol level in PCS patients drawn according to results of the phasic alertness test and the values of the controls in different brain regions. Myo‐inositol (mI) level categorized for post‐COVID syndrome (PCS) patients with normal results in phasic alertness (PCS norm, orange, *n* = 23), with pathological results in phasic alertness (PCS path, red, *n* = 7) from the test battery for the assessment of attention (TAP) and the values of the control group (C, blue, *n* = 30). ANCOVA and post hoc test were used for statistical analysis. CBL, cerebellum; LFL, left frontal lobe; LOL, left occipital lobe; LPL, left parietal lobe; LTL, left temporal lobe; RFL, right frontal lobe; ROL, right occipital lobe; RPL, right parietal lobe; RTL, right temporal lobe.

**Figure 4:**
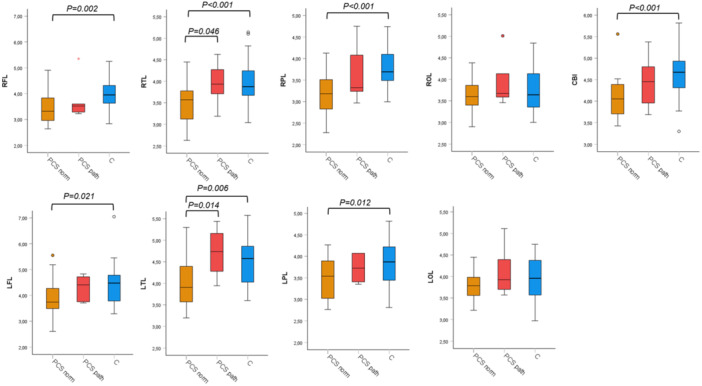
Myo‐inositol level in PCS patients drawn according to results of the flexible reaction time test and the values of the controls in different brain regions. Myo‐inositol (mI) level; categorized for post‐COVID syndrome (PCS) patients with normal results in flexible reaction time (PCS norm, orange, *n* = 24), with pathological results in flexible reaction time (PCS path, red, *n* = 6) from the test battery for the assessment of attention (TAP) and the values of the control group (C, blue, *n* = 30). ANCOVA and post hoc test were used for statistical analysis. CBL, cerebellum; LFL, left frontal lobe; LOL, left occipital lobe; LPL, left parietal lobe; LTL, left temporal lobe; RFL, right frontal lobe; ROL, right occipital lobe; RPL, right parietal lobe; RTL, right temporal lobe.

While the paired *t*‐test between PCS patients and healthy controls showed significantly decreased brain mI levels in the patients in multiple brain regions, the comparison between patients with and those without certain abnormal psychometric test results using ANCOVA revealed no significant differences, though there was a tendency of higher mI levels in those with impaired phasic alertness and flexible reaction time compared to those without, and remarkably a tendency of lower mI levels in those with impaired figure recognition in the WFMT compared to those without (Figures 2, 3, 4). The presence or absence of characteristic PCS symptoms did not result in significant differences in brain mI levels between the patient subgroups. Details are shown in Supporting Information S1–S4: Figures [Link], [Link], [Link], [Link].

The spectral linewidth did not significantly differ between the patient subgroups defined according to their PCS symptoms or psychometric test results. The Spearman rank test did not reveal significant correlations between altered brain metabolite concentrations and the neuropsychological findings in the patients.

## 4 Discussion

We present wbMRSI data of 30 PCS patients examined in median of 14 months after SARS‐CoV‐2 infection in comparison to data of 30 healthy controls matched for age, sex, and BMI—factors that are considered to substantially affect metabolite levels [31, 32, 33]. Using wbMRSI, metabolic changes were for the first time simultaneously assessed within the whole brain of the patients. The derived mean metabolite values over each of the eight brain lobes as well as over cerebellum enable us a clear overview about the PCS‐related brain metabolic changes, where the main finding was significantly decreased myo‐inositol levels bilaterally in the frontal, temporal, and parietal lobes and in the cerebellum of the patients, with an accentuation in the right hemisphere. In addition, decreased Glx in the right parietal lobe and increased tCr in the left frontal lobe as well as a significant increase in linewidth in the frontal, temporal, and parietal lobes were observed in the patients compared to controls.

A strength of the study is the well‐defined cohort of patients and controls matched for age, sex, and BMI. We adhered to strict exclusion criteria to make the study group homogeneous. Another strength is the use of wbMRSI, a recently established MR spectroscopic method allowing to measure brain metabolites within the whole brain with high spatial resolution [18, 19, 20]. Using this method, we could detect PCS‐associated brain metabolic alterations nearly within the whole brain.

In the present study, we focused on getting an overview about the PCS‐associated metabolic changes within the whole brain, therefore, we determined the mean metabolite values over lobar structures as well as over the cerebellum without considering possible inhomogeneous distribution of the metabolites within brain lobes. Further studies addressing this limitation are planned to study metabolite changes as well as multiparametric MRI changes in specific small brain structures in detail. This will allow to obtain more insight into PCS‐associated alterations.

Former MRS studies of the brain in PCS patients focused upon γ‐aminobutyric acid (GABA) or glutamate/glutamine (Glx) levels in the patients compared to controls [34, 35]. Saleh et al. [34] report reduced glutathione levels but no changes in GABA levels in the frontal gray and white matter in the patients. They conclude that PCS is associated with ongoing oxidative stress in the brain [34]. Sklinda et al. [35] found increased Glx levels and slightly reduced lactate levels in the deep gray matter in PCS patients with brain fog compared to controls. Marinkovic et al. [36] observed a decrease of GABA+/water in the occipital cortex of the patients compared to controls, and in tendency lower NAA levels. None of the three studies reported myo‐inositol levels.

To the best of our knowledge, mI alterations were only once before described in PCS patients. In agreement with our findings, Ernst et al. [17] found reduced mI levels in the anterior cingulate cortex–gray matter of patients with PCS. In contrast to the present study, they measured brain metabolites from only two voxels—one in the frontal white matter (FWM) and one located in the anterior cingulate cortex–gray matter (ACC–GM). In addition to mI alteration, they also found lower NAA levels in both regions, as well as lower glutamate and glutamine in FWM. The discrepancies between the studies may indicate a rather inhomogeneous distribution of the brain metabolites except for the glial marker mI indicating nearly ubiquitous glial alterations in the patients.

Myo‐inositol (mI)—a sugar alcohol and main player in the inositol family—is found predominantly in astrocytes. It is considered a marker for glial cell activation and neuroinflammation [37, 38]. Myo‐inositol increases have been observed in several neuroinflammatory diseases [32, 39], but also in neurodegenerative diseases with a chronic pro‐inflammatory state, such as Alzheimer's disease, for example [40]. An increase of mI was also observed with increasing BMI and with age [31, 32] and in patients with manic and hypomanic episodes in Bipolar disorder [41]. Of note, using 11C‐PK11195‐PET, microglia activation has been shown in patients with HCV encephalopathy in the same regions as the mI increase. Moreover, microglia activation was associated in the respective study with less cognitive impairment [42].

A decrease in cerebral mI levels according to proton MRS studies was observed in patients with liver cirrhosis and hepatic encephalopathy (HE) [43], in patients with hyponatremia [43], in patients with thyreotoxicosis [44], and in patients with major depression or schizophrenic psychosis [41]. The finding in patients with HE, as well as in hyponatremia, was explained by the role of mI as an osmolyte. Neither the findings in the psychiatric patients, nor the findings in patients with thyreotoxic crisis could be explained. It was noted, however, that they were reversible with successful treatment [41, 44].

Myo‐inositol increases are usually considered to represent both activated astrocytes and microglia. Indeed, neuroinflammation is characterized by a cross‐talk between these two cell types. Neuron‐glia interactions are modulated by immunological signals [5]. Microglia may secrete both pro‐inflammatory as well as anti‐inflammatory molecules that regulate astrocytic activity. On the other hand, astrocytes may regulate microglia activity via production of activating or inhibiting mediators, such as cytokines, growth factors, or complement, for example [45]. Thereby, microglia and astrocytes can change their phenotypes depending upon their environment—from a pro‐inflammatory state to a resting or to an anti‐inflammatory state. It is assumed that in case of chronic infection and consecutive chronic activation, microglia may become dysfunctional and resistant to normal regulation [46].

Several autopsy studies have shown an over‐reactivity of astrocytes and microglia in the subcortical white matter in patients who died with COVID‐19 [47, 48]. Similar findings are reported from animal experiments [47, 49, 50]. This overactive state of microglia is supposed to lead to a decrease in hippocampal neurogenesis as well as the death of myelinating oligodendrocytes [51]. Samples from patients who died with PCS are not available. Thus, the microglia reaction to the SARS‐CoV‐2 infection in the long term is not known.

Following the hypothesis of a dysregulated CNS immune response to the virus, with ongoing microglia and astrocyte activation, an increase in myo‐inositol levels would be expected. Instead, we observed a decrease in the patients' myo‐inositol levels in the frontal, temporal, and parietal lobes as well as in the cerebellum. Patients with deficits in the figural memory according to the WFMT showed even lower mI levels than those without, especially in the left temporal and parietal lobes. Due to the small number of patients in this group, however, the difference between the patient groups did not reach the level of significance in an ANCOVA considering age and sex as cofactors. Of interest, the patients with deficits regarding phasic alertness and flexibility, according to their TAP results, showed higher mI levels than those without, although both groups had lower mI levels than the controls. Though these results must be considered cautiously regarding the small number of patients, there is one interesting observation that deserves further study. All but one of the patients with the memory deficit are female, and all but one of those with attention deficits are male. Of interest mI levels were lower in the female compared to the male patients, while there was no difference between male and female controls (Supporting Information S5: Figure 5). So far, it is not clear whether the divergent findings regarding mI alterations in patients with memory or attention deficits could be sex related, a fact that would further support the clinical observation that females are more susceptible for the pathologies behind PCS than males.

In general, it could be hypothesized that the glial hyperactivity induced by the SARS‐CoV‐2 infection and maintained by the systemic chronic inflammatory response might finally lead to an exhaustion of both astrocytes and microglia that is represented by a decrease of the mI content.

Our results are in line with those observed by a recent ^18^F‐FDG‐PET study in 28 PCS patients performed 16.4 ± 5.9 months after COVID‐19 compared to age and sex matched healthy controls. This study showed significant hypometabolism in the right frontal and the temporal lobes. Longer duration of symptoms was associated with lower metabolism [14]. Of note, this patient group resembles that in our present study regarding COVID‐19 severity, age, sex distribution and time interval since the SARS‐CoV‐2 infection, and the cerebral glucose utilization represents, to a huge extent, glial metabolism since glial cells predominate in the brain.

The decrease in myo‐inositol levels in our patients with PCS is in line with our findings in patients with HCV encephalopathy [39], where we observed a negative correlation between the fatigue score (FIS) and myo‐inositol levels in the parieto‐occipital white matter.

In the present study, none of the other metabolites assessed showed significant differences between patients and controls. On the lobar level, we had neither indication for alteration of neuronal integrity considering NAA, nor indications for alterations of cellular membrane turnover or alterations of cerebral energy metabolism considering Cho and tCr. Considering especially the unaltered Cho levels, we would not interpret the decrease of mI as an osmolytic response similar to the alterations in patients with liver cirrhosis [52]. The unaltered choline levels also argue against structural damage of astrocytes. It must be emphasized, however, that our findings do not exclude cell damage in delimited brain regions, as these limited damages may remain undetected considering the metabolites in the different lobes.

The second finding of our study refers to linewidth. Linewidth is a measure of the homogeneity of the spectra. An increase of linewidth has been observed with age and with increasing BMI, for example [33, 53]. Moreover, spectral linewidth differs between male and female subjects [36]. Since patients and controls are matched for age, sex, and BMI in this study, the difference in linewidth between patients and controls suggests disease‐related structural alterations of the respective brain regions or alterations of perfusion. Of note, hypoperfusion affecting the frontal, parietal, and temporal cortex in PCS patients has recently been shown using arterial spin labeling MRI [12]. Another possible cause of the linewidth difference could be a difference in tissue water concentration between the groups [33]. Indeed, Petersen et al. [11] observed a significant increase in extracellular free water in all brain lobes in a diffusion MRI study of 223 subjects in median 289 days after SARS‐CoV‐2 infection compared to data of a control group that had been studied before the pandemic. These alterations were present although the patients had no clinical symptoms of PCS. A further possible reason could be increased brain paramagnetic depositions like iron in our patients [54], however so far, there are no indications of such deposits.

To conclude, a nearly generalized decrease of brain myo‐inositol levels is evident in our PCS patients, suggesting a long‐lasting influence of the SARS‐CoV‐2 infection on their brain immunoactivity. A functional downregulation of astrocytes may be a possible explanation. Further studies are needed to prove the hypothesis. Moreover, it is important to acknowledge that wbMRSI is only a supplement tool to structural or functional MR imaging, albeit it provides valuable and sensitive insight into metabolic alterations that are not captured by conventional MRI techniques. A combined analysis of structural alterations assessed by multimodal MRI and the accompanying metabolic changes according to wbMRSI in distinct functionally well‐characterized specific brain regions in further study is necessary to bring more insight into the PCS‐related brain pathology.

## Author Contributions

Ann‐Katrin Hennemann and Nima Mahmoudi contributed equally to this work as the first authors. Meike Dirks and Xiao‐Qi Ding are joint senior authors. Karin Weissenborn, Xiao‐Qi Ding, Heinrich Lanfermann, and Meike Dirks designed the study. Meike Dirks, Ann‐Katrin Hennemann, and Nima Mahmoudi had a major role in the acquisition of data. Meike Dirks, Karin Weissenborn, Ann‐Katrin Hennemann, Xiao‐Qi Ding, Nima Mahmoudi, and Katja Döring analyzed and interpreted the data. Karin Weissenborn and Xiao‐Qi Ding wrote the first draft of the report. All authors interpreted the findings, critically reviewed, and revised the report, and had final approval of the submitted paper. The corresponding author (Meike Dirks) attests that all listed authors meet authorship criteria and that no others meeting the criteria have been omitted.

## Ethics Statement

This study was carried out in accordance with the Declaration of Helsinki. The study was approved by the Local Ethics Committee (Nr. 10093_B0_S_2021).

## Consent

All participants included in the study gave written informed consent.

## Conflicts of Interest

The authors declare no conflicts of interest.

## Supporting information


**Supporting Figure 1:** Memory impairment revised.

**Supporting Figure 2:** Concentration deficits revised.

**Supporting Figure 3:** Sleep disturbances revised.

**Supporting Figure 4:** Difficulties finding words revised.

**Supporting Figure 5:** mI level gender final.

Supporting material Figure legend MRT PCS revised clear final.

## Data Availability

Anonymized data are available on reasonable request from the corresponding author at dirks.meike@mh-hannover.de.
