# Supplementary figures and images for: Evidence of Impaired Neuroimmune System in Post‐COVID Syndrome—A Whole Brain Magnetic Resonance Spectroscopy Study
Source: J Med Virol. 2025 Dec 22;97(12):e70762. doi: 10.1002/jmv.70762 (PMC12720396; doi:10.1002/jmv.70762)

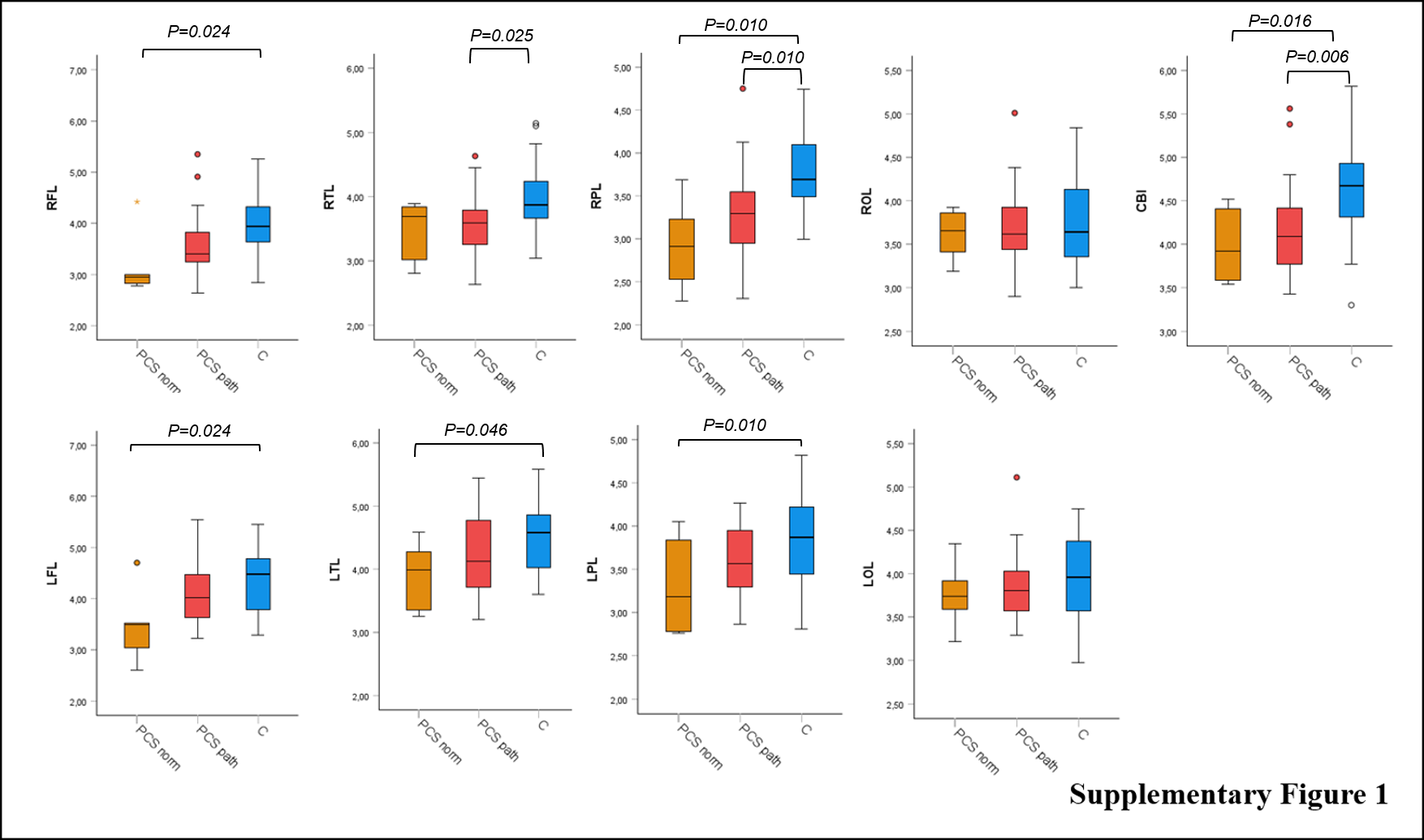

Supplement: Supplementary file 1 — Supporting Figure 1: Memory impairment revised. [file JMV-97-e70762-s004.tif]

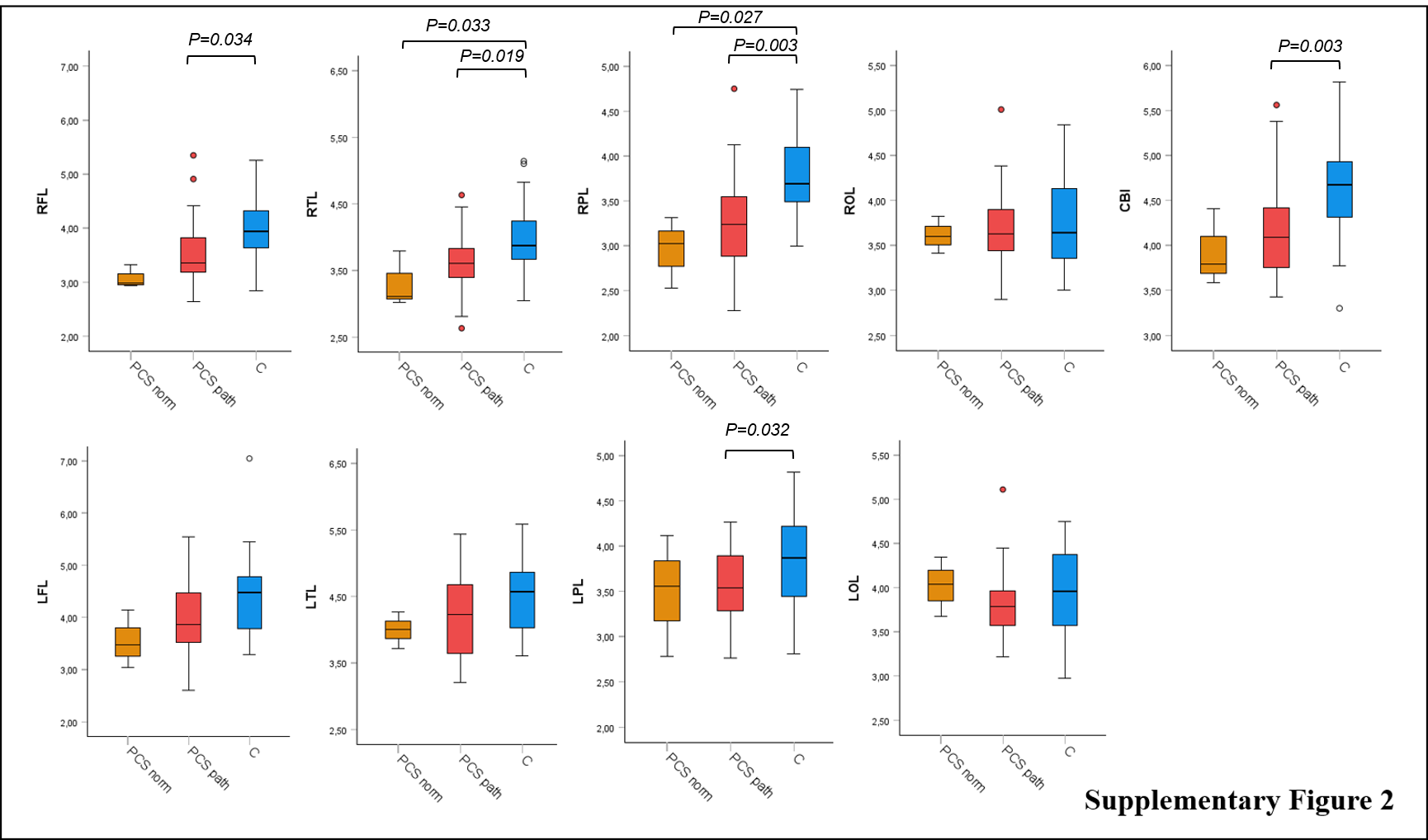

Supplement: Supplementary file 2 — Supporting Figure 2: Concentration deficits revised. [file JMV-97-e70762-s003.tif]

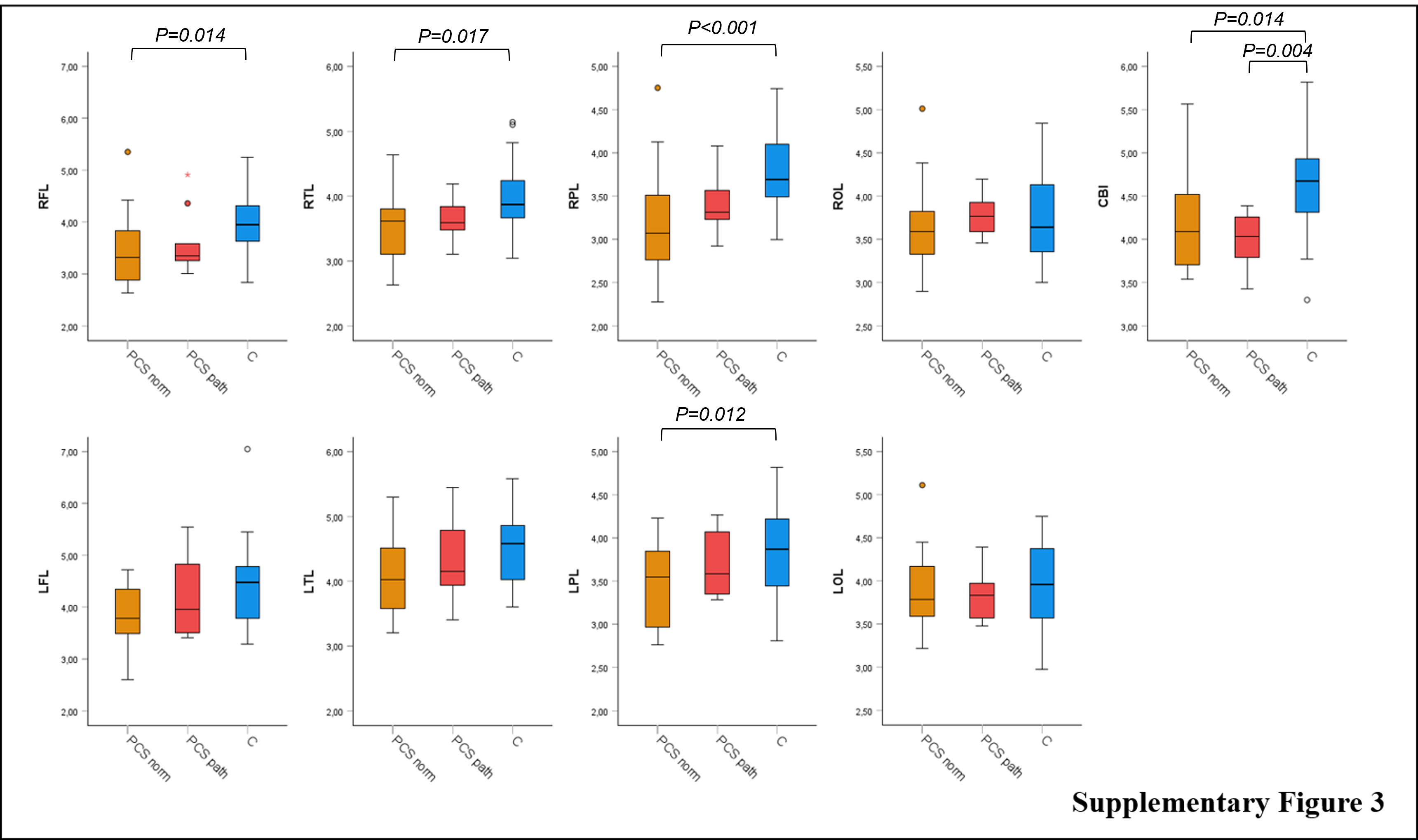

Supplement: Supplementary file 3 — Supporting Figure 3: Sleep disturbances revised. [file JMV-97-e70762-s006.tif]

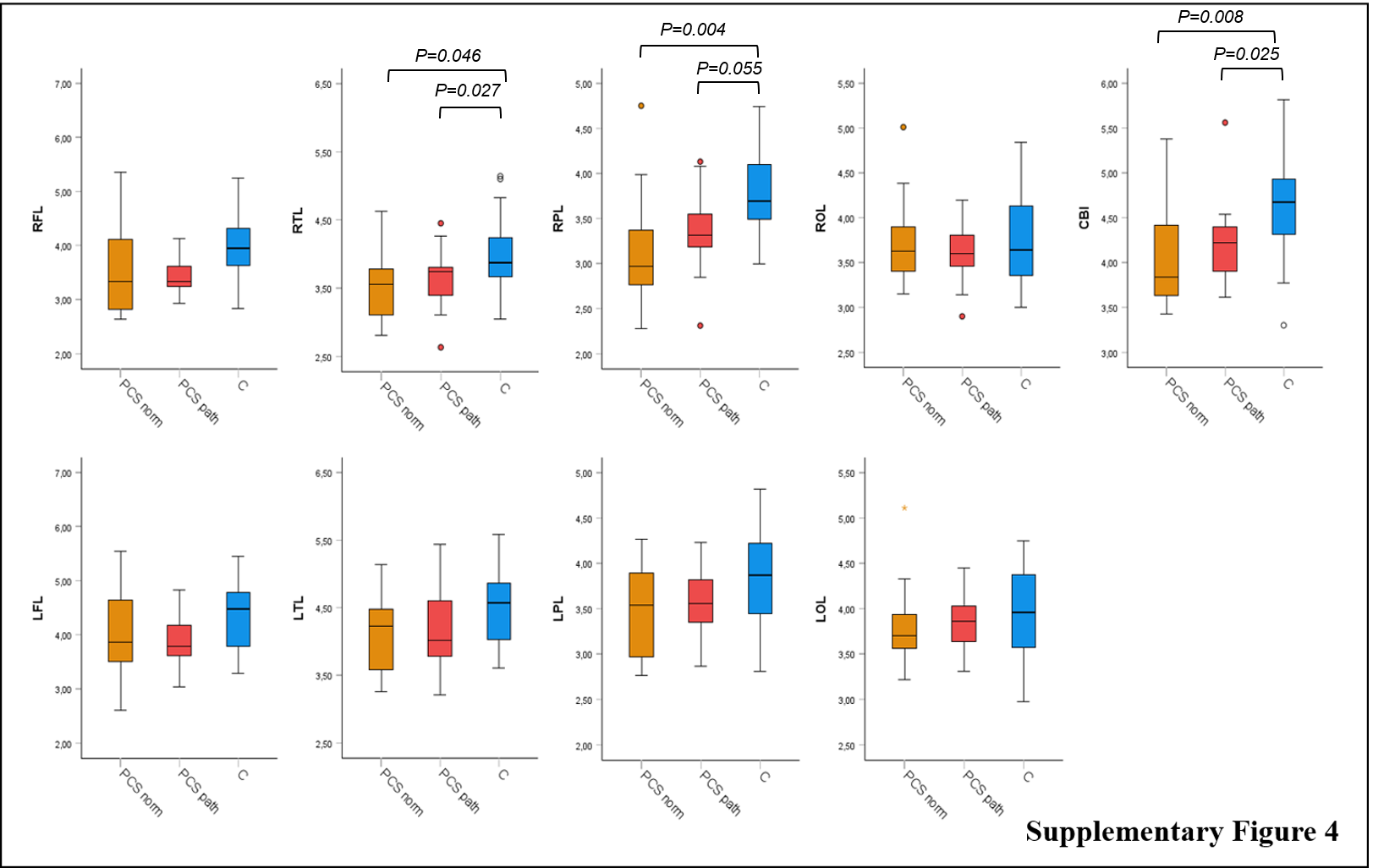

Supplement: Supplementary file 4 — Supporting Figure 4: Difficulties finding words revised. [file JMV-97-e70762-s001.tif]

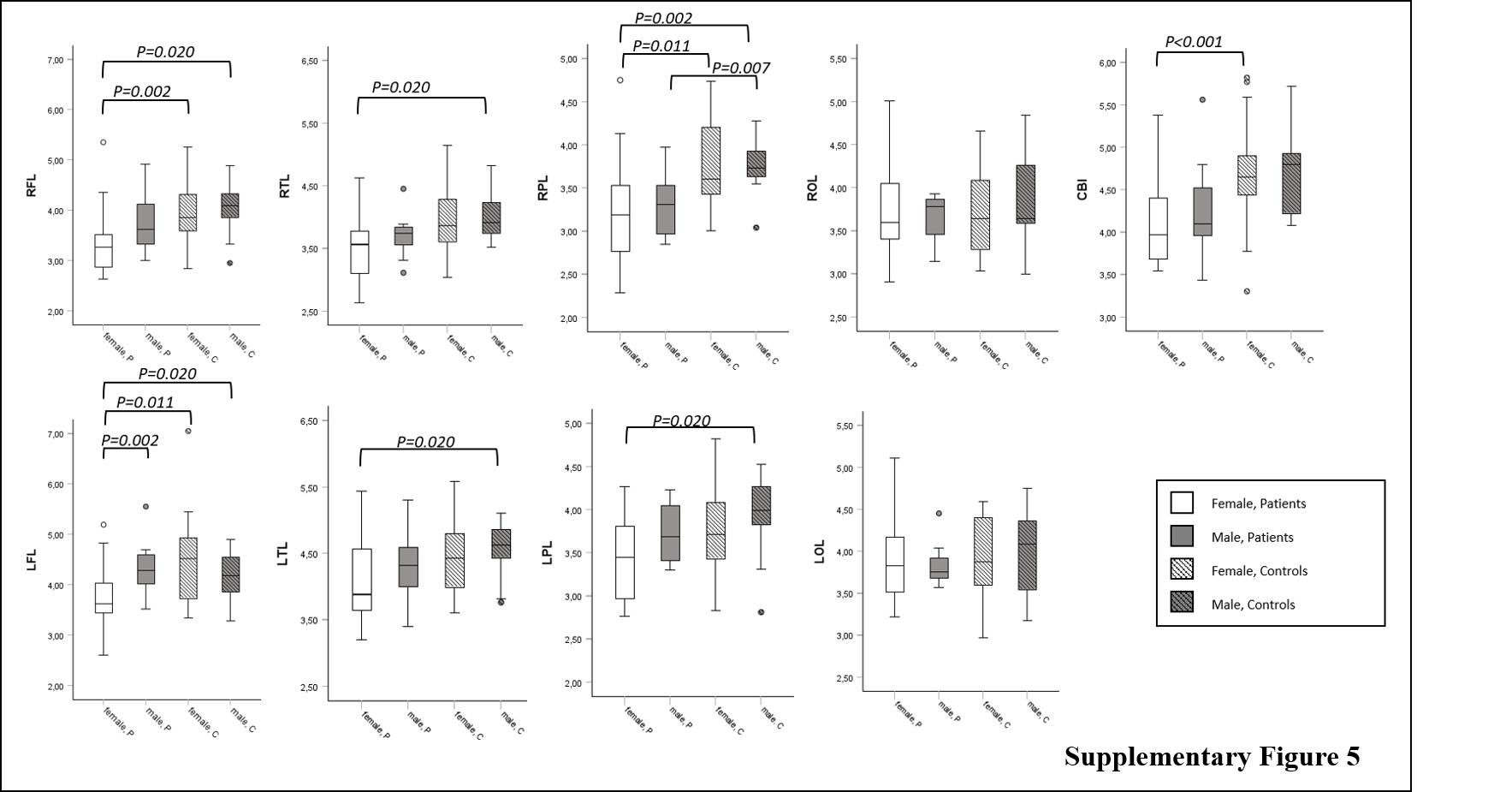

Supplement: Supplementary file 5 — Supporting Figure 5: mI level gender final. [file JMV-97-e70762-s005.tif]
