# Supplementary material for: Evidence of Impaired Neuroimmune System in Post‐COVID Syndrome—A Whole Brain Magnetic Resonance Spectroscopy Study
Source: J Med Virol. 2025 Dec 22;97(12):e70762. doi: 10.1002/jmv.70762 (PMC12720396; doi:10.1002/jmv.70762)
Supplement: Supplementary file 6 — Supporting material Figure legend MRT PCS revised clear final. [file JMV-97-e70762-s002.docx]

**Evidence of impaired neuroimmune system in Post-COVID syndrome – a whole brain Magnetic Resonance Spectroscopy study**

Running title: **Whole brain MRS in Post-COVID-Syndrome**

Ann-Katrin Hennemann^1^, Nima Mahmoudi^2^, Katja Döring^2^, Heinrich Lanfermann^2^, Karin Weissenborn^1^, Meike Dirks^1^*, Xiao-Qi Ding^2^

^1^Department of Neurology, Hannover Medical School, 30625 Hannover, Germany

^2^ Institute of Diagnostic and Interventional Neuroradiology, Hannover Medical School, 30625 Hannover, Germany

**Supplementary Material**

**Figure legends**

**Supplementary Fig. 1 Myo- inositol level in PCS patients according to the presence or absence of memory impairment and controls in different brain regions**

Myo Inositol (mI) level; categorized for post-covid syndrome (PCS) patients with no memory impairment (PCS norm, orange, n=6), with memory impairment (PCS path, red, n=24) and control group (C, blue, n=30). Analysis of Covariance (ANCOVA) including age and sex as covariates and Bonferroni correction for multiple comparisons were used for statistical analysis.

RFL, right frontal lobe; LFL, left frontal lobe; RTL, right temporal lobe; LTL, left temporal lobe; RPL, right parietal lobe; LPL, left parietal lobe; ROL, right occipital lobe, LOL, left occipital lobe; CBL, cerebellum

**Supplementary Fig. 2 Myo- inositol level in PCS patients according to the presence or absence of concentration deficits and controls in different brain regions**

Myo Inositol (mI) level; categorized for post-covid syndrome (PCS) patients with no concentration deficits (PCS norm, orange, n=3), with concentration deficits (PCS path, red, n=27) and control group (blue, n=30); Analysis of Covariance (ANCOVA) including age and sex as covariates and Bonferroni correction for multiple comparisons were used for statistical analysis

RFL, right frontal lobe; LFL, left frontal lobe; RTL, right temporal lobe; LTL, left temporal lobe; RPL, right parietal lobe; LPL, left parietal lobe; ROL, right occipital lobe, LOL, left occipital lobe; CBL, cerebellum

**Supplementary Fig. 3 Myo- inositol level in PCS patients according to the presence or absence of sleep disturbances and controls in different brain regions**

Myo Inositol (mI) level; categorized for post-covid syndrome (PCS) patients with no sleep disturbances (PCS norm, orange, n=20), with sleep disturbances (PCS path, red, n=10) and control group (C, blue, n=30). Analysis of Covariance (ANCOVA) including age and sex as covariates and Bonferroni correction for multiple comparisons were used for statistical analysis.

RFL, right frontal lobe; LFL, left frontal lobe; RTL, right temporal lobe; LTL, left temporal lobe; RPL, right parietal lobe; LPL, left parietal lobe; ROL, right occipital lobe, LOL, left occipital lobe; CBL, cerebellum

**Supplementary Fig. 4 Myo- inositol level in PCS patients according to the presence or absence of difficulties in finding words and controls in different brain regions**

Myo Inositol (mI) level; categorized for post-covid syndrome (PCS) patients with no difficulties in finding words, (PCS norm, orange, n= 15), with difficulties in finding words (PCS path, red, n= 15) and control group (C, blue). Analysis of Covariance (ANCOVA) including age and sex as covariates and Bonferroni correction for multiple comparisons were used for statistical analysis.

RFL, right frontal lobe; LFL, left frontal lobe; RTL, right temporal lobe; LTL, left temporal lobe; RPL, right parietal lobe; LPL, left parietal lobe; ROL, right occipital lobe, LOL, left occipital lobe; CBL, cerebellum

**Supplementary Fig. 5 Myo- inositol level in female and male post-covid-syndrome (PCS) patients vs. female and male controls in different brain regions**

Kruskal-Wallis Test and post hoc test were used for statistical analysis.

Abbreviations: RFL, right frontal lobe; LFL, left frontal lobe; RTL, right temporal lobe; LTL, left temporal lobe; RPL, right parietal lobe; LPL, left parietal lobe; ROL, right occipital lobe, LOL, left occipital lobe; CBL, cerebellum
